# Enhanced Phosphate Capture by Thermally Modified Calcium Aluminate Decahydrate: Optimization, Performance and Mechanism

**DOI:** 10.3390/molecules31122174

**Published:** 2026-06-21

**Authors:** Peng Cheng, Ruixiang Wang, Yu Liu, Yu Shang, Lei Yang, Yong-Xiang Ren

**Affiliations:** 1 Nanyang Key Laboratory of Water Pollution Control and Solid Waste Resource Recovery, Nanyang Institute of Technology, Nanyang 473004, China; wangruixiang35@163.com (R.W.); gps_liuyu@126.com (Y.L.); yshangnyist@163.com (Y.S.); 2 School of Civil Engineering, Nanyang Institute of Technology, Nanyang 473004, China; 3 Shaanxi Key Laboratory of Environmental Engineering, Xi’an University of Architecture and Technology, Xi’an 710055, China; yangleigps@xauat.edu.cn (L.Y.); ryx@xauat.edu.cn (Y.-X.R.)

**Keywords:** adsorption, phosphorus, thermally modification, calcium aluminate

## Abstract

Adsorption is a promising technology for phosphate removal to alleviate eutrophication. In this study, thermally modified calcium aluminate decahydrate (TCAH) was prepared via low-temperature thermal treatment of calcium aluminate decahydrate (CAH_10_) to develop a cost-effective and high-performance phosphate adsorbent. The optimal modification temperature was determined to be 120 °C, which reduced the crystallinity of CAH_10_, enhanced its porosity, and induced the formation of amorphous calcium aluminate phases. Batch adsorption experiments showed that TCAH exhibited a maximum adsorption capacity of 199.80 mg P/g at 25 °C. The adsorption kinetics followed the pseudo-second-order model, while the adsorption isotherms were well fitted by the Redlich–Peterson model. TCAH maintained high removal efficiency over a wide pH range of 3.0–11.0 and showed high selectivity against common coexisting anions. Characterizations using SEM-EDS, XRD, FTIR and XPS suggested that phosphate removal by TCAH was dominated by synergistic amorphous precipitation and inner-sphere complexation. In tests with real phosphorus-releasing liquor derived from excess sludge, TCAH achieved nearly complete phosphate removal at a dosage of 5 g/L within 6 h. Owing to its readily available raw materials, low preparation temperature, and outstanding phosphate capture performance, TCAH is a promising candidate for efficient phosphate capture and recovery from wastewater.

## 1. Introduction

Phosphorus (P) is one of the primary contributors for eutrophication in water bodies, which can lead to detrimental algal blooms, reduced dissolved oxygen, generation of toxic substances, and even the collapses of drinking water supply systems [1]. Reducing the excessive discharge of P in wastewater and lowering its concentration in water bodies are crucial for controlling water eutrophication. Furthermore, even a P concentration of 0.02 mg P/L is too high for some sensitive water bodies [2,3]. Therefore, various technologies have been developed for phosphate removal, and increasingly stringent P discharge standards have been implemented [4].

Among the methods for phosphate removal, adsorption exhibits promising application prospects due to its design flexibility, operational simplicity, low chemical consumption, and high removal efficiency at low phosphate concentrations [4]. Therefore, an increasing number of novel adsorbents were developed to promote the real application of phosphate adsorption, such as modified La-MOF [5], Zr-MOFs [6], ZIF-8@aminated PAN-PVP material [7], Lanthanum functionalised forestry waste biochar [8], La-Zr co-modified hydrogel beads [9], and so on.

Due to the strong specific affinity and adsorption selectivity for phosphate, metal (hydr)oxide-based adsorbents have been extensively studied, such as those containing aluminum (Al), calcium (Ca), zirconium (Zr), cerium (Ce), and lanthanum (La) [10,11]. Nevertheless, most reported adsorbents are challenged by large-scale fabrication. Such materials generally remain confined to the lab-scale stage because of the costly rare-earth raw materials [4,12]. Moreover, various functional adsorbents require complicated synthesis procedures, such as pyrolysis, impregnation, electrochemical modification and hydrothermal synthesis [13,14], which further restrict the widely application of adsorption.

In our previous study, commercially available calcium aluminate was directly hydrated to synthesize CAH_10_ (CaO·Al_2_O_3_·10H_2_O, abbreviated as CAH_10_) as a readily prepared adsorbent [15]. The structure with ring-shaped units of three edge-sharing Ca(OH)_6_(H_2_O)_2_ polyhedra endowed CAH_10_ with prominent phosphate adsorption performance at low phosphate concentrations [16]. However, its maximum adsorption capacity and initial adsorption rate are still inferior to those of state-of-the-art novel adsorbents. Previous studies have confirmed that thermal treatment can induce crystal transformation in CAH_10_, reduce its density, and increase the porosity [17]. Furthermore, thermal treatment could also generate amorphous aluminate structures with strong potential for phosphate adsorption [11,18,19]. Hence, thermally modified CAH_10_ is a remarkable potential candidate for phosphate adsorption.

Accordingly, CAH_10_ was thermal modified to develop a novel phosphate adsorbent (thermally modified calcium aluminate decahydrate, abbreviated as TCAH). Specifically, this study aims to: (1) conduct a comparative analysis of the changes in pore distribution, crystal structure, and other properties of CAH_10_ following thermal modification; (2) determine the optimal modification temperature and evaluate the adsorption performance; (3) elucidate the phosphate removal mechanism and assess potential for practical application.

## 2. Results and Discussion

### 2.1. Characterization of Thermally Modified CAH_10_

**Figure 1:**
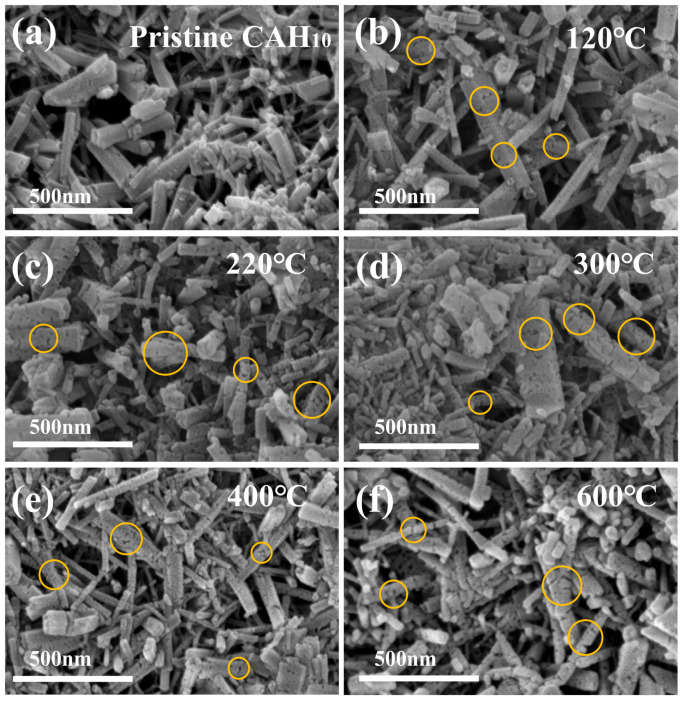
SEM images of pristine CAH_10_ (**a**) and thermally modified CAH_10_ (**b**–**f**). (Modified temperature = 120 °C (**b**), 220 °C (**c**), 300 °C (**d**), 400 °C (**e**) and 600 °C (**f**); yellow circle: pores and cracks on the crystal surface).

Figure 1 shows the SEM images of pristine CAH_10_ and thermally modified CAH_10_. As presented in Figure 1a, the pristine CAH_10_ exhibited relatively intact prismatic crystals. After thermal modification, several pores were visible on the surface of the prismatic crystals, as marked by yellow circles in Figure 1b–f, indicating the dehydration or decomposition of CAH_10_. As the modification temperature increased from 120 °C to 600 °C, the number of pores on the prisms gradually increased, accompanied by the appearance of minor cracks. The pores and cracks on the prismatic crystals increased the porosity of the material and enhanced its phosphate adsorption potential.

**Figure 2:**
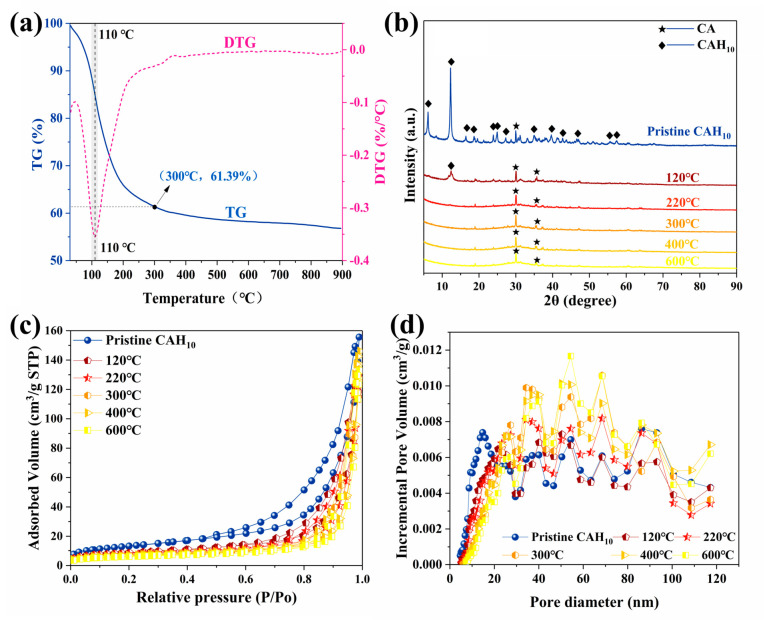
Thermogravimetric analysis of CAH_10_ (**a**), XRD patterns (**b**), N_2_ adsorption/desorption isotherms (**c**), and the pore size distributions based on BJH method (**d**) of pristine CAH_10_ and thermally modified CAH_10_. (Modified temperature = 120 °C, 220 °C, 300 °C, 400 °C and 600 °C).

The thermogravimetric (TG) and derivative thermogravimetric (DTG) curves of CAH_10_ are shown in Figure 2a. The weight loss rate of CAH_10_ reached a maximum at approximately 110 °C. CAH_10_ began to decompose at approximately 65 °C, possibly producing C_3_AH_6_ (3CaO·Al_2_O_3_·6H_2_O) and gibbsite (Al_2_O_3_·3H_2_O) [20]. Subsequently, further dehydration of gibbsite may occur in the temperature range of 200–300 °C. As presented in Figure 2a, CAH_10_ exhibited a total weight loss of 38.61% at 300 °C, which was approximately consistent with the theoretical values calculated from previous research [21].

The XRD patterns in Figure 2b indicate that thermal modification at all tested temperatures did not result in crystallization of C_3_AH_6_ or gibbsite. Instead, the thermal treatment significantly weakened the crystallinity of CAH_10_ and led to the formation of an amorphous phase. Meanwhile, as observed from the micromorphology of the materials shown in Figure 1, the overall prismatic framework of the material remained unchanged with reduced crystallinity by the thermal modification.

As shown in Figure 2c, the nitrogen adsorption/desorption capacity of the material gradually decreased with increasing modification temperature. Figure 2d shows that increasing the thermal modification temperature generally led to a significant increase in the proportion of mesopores and macropores. Based on the pore volume and pore diameter data (see Table S1 in the Supplementary Material), the average pore diameter increased significantly with the rise in thermal modification temperature. This is consistent with the SEM (Figure 1) and pore size distribution (Figure 2d) results.

Figure S1 shows the FTIR spectra of CAH_10_ after thermal modification at various temperatures. The broad absorption band centered at approximately 3550 cm^−1^ could be ascribed to O-H stretching vibrations, likely associated with adsorbed H_2_O, hydroxides, or hydrates [22,23]. The absorption peaks observed at around 1060 cm^−1^ and 1414 cm^−1^ could be ascribed to Al=O and Al-H stretching vibrations, respectively [24]. The band at 532 cm^−1^ could be assigned to the lattice vibration of (Ca, Al)-O [25]. After modification at 120 °C, the intensity of the absorption peak at 532 cm^−1^ decreased, and the peak disappeared completely at higher modification temperatures. A new absorption peak emerged near 792 cm^−1^ after modification, which may also be related to (Ca, Al)-O lattice vibrations [25,26]. Overall, the thermal modification altered certain surface functional groups of CAH_10_, which is consistent with the XRD and SEM analysis.

### 2.2. Phosphate Adsorption on Thermally Modified CAH_10_

**Figure 3:**
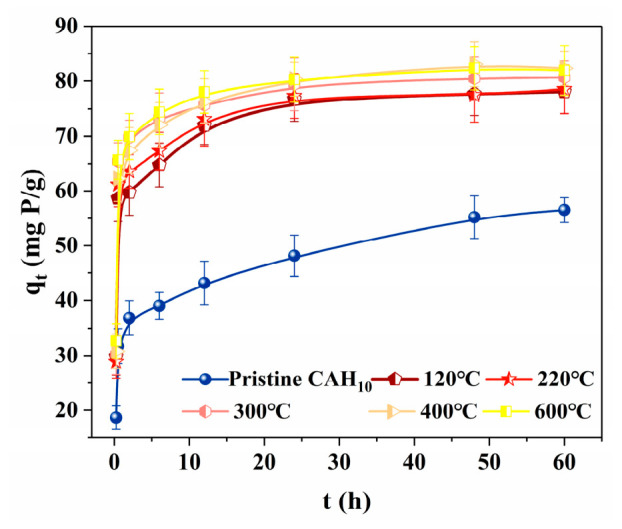
Phosphate adsorption on pristine CAH_10_ and thermally modified CAH_10_. (Modified temperature = 120 °C, 220 °C, 300 °C, 400 °C, and 600 °C, adsorption dose = 0.1 g/L, C_0_ = 10 mg P/L, T = 25 °C, pH = 7.0, contact time = 60 h).

Figure 3 presents the phosphate adsorption on pristine CAH_10_ and thermally modified CAH_10_ at different temperatures. Compared with pristine CAH_10_, thermal modification significantly enhanced the phosphate adsorption capacity. After thermal modification at 120 °C, the material achieved an adsorption capacity of approximately 78.10 mg P/g within 60 h. Meanwhile, the adsorption capacity at 0.5 h reached 58.63 mg P/g, indicating a significant increase in the initial adsorption rate.

When the thermal modification temperature was further increased from 120 °C to 600 °C, only a marginal increase in adsorption capacity was observed. The phosphate adsorption capacity of the material modified at 600 °C was 81.96 mg P/g within 60 h, which was only 4.94% higher than that modified at 120 °C, indicating that thermal modification above 120 °C had no remarkable enhancement on the adsorption performance. Overall, thermal modification at 120 °C significantly enhanced both the adsorption capacity and initial adsorption rate, while further increasing the modification temperature had a rather limited effect. This may be attributed to the fact that the phosphate adsorption performance is influenced not only by specific surface area and porosity but also by changes in crystal structure.

Accordingly, thermal modification at 120 °C already lead to an amorphous structure (Figure 2b) and a porous morphology (Figure 1b). Although raising the temperature to 600 °C further increased the average pore diameter (Table S1) and slightly improved the adsorption capacity from 78.10 to 81.96 mg P/g (+4.94%), the BET surface area decreased from 50.8 to 32.2 m^2^/g. This suggested that the amorphous phase formed at 120 °C provided sufficient active sites for phosphate capture, and the moderate porosity was more decisive for the overall adsorption performance than the specific surface area alone. Considering the need for energy conservation and adsorption performance optimization, CAH_10_ thermally modified at 120 °C was selected for subsequent tests in this study, which is denoted as TCAH hereinafter.

### 2.3. Adsorption Kinetics, Isotherms, and Thermodynamics

**Figure 4:**
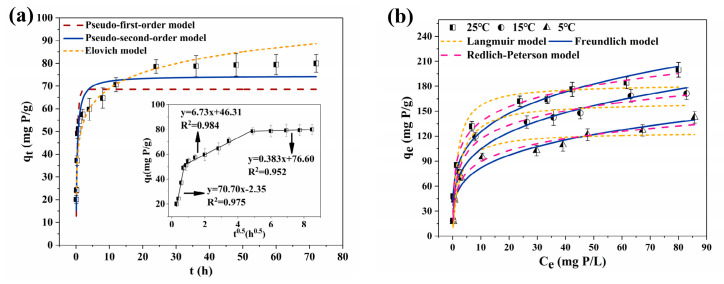
Phosphate adsorption kinetics (**a**) and adsorption isotherms (**b**) on TCAH; (**a**) inset: intra-particle diffusion model fitting result. (Adsorbent dose = 0.1 g/L, pH = 7.0, C_0_ = 10 mg P/L in (**a**), C_0_ = 2–100 mg P/L in (**b**)).

The effect of contact time on phosphate adsorption by TCAH is shown in Figure 4a. During the initial stage (0–0.5 h), the adsorption capacity increased rapidly to 49.16 mg P/g. Subsequently, the adsorption rate began to decelerate, and adsorption equilibrium was reached after approximately 24 h, with an adsorption capacity of approximately 78.59 mg P/g.

The pseudo-first-order, pseudo-second-order, Elovich, and intra-particle diffusion kinetic models were employed to describe the adsorption kinetics results (see Text S1 in the Supplementary Material). As presented in Figure 4a and Table S2, the pseudo-second-order kinetic model exhibited a higher R^2^ and a lower χ^2^, indicating that the removal rate was proportional to the square of the number of possible unoccupied sites on the surface of the adsorbent [27]. The increased pore diameter and active sites of materials may also contribute to the quick kinetics.

The inset in Figure 4a shows the fitting results of the intra-particle diffusion model. The phosphate adsorption by TCAH can be divided into three stages: 0–0.5 h, 0.5–24 h, and after 24 h. The adsorption was rapid in the first stage, slowed down obviously in the second stage, and reached equilibrium in the final stage. The linear fitting coefficients (R^2^) of the three stages were 0.975, 0.984, and 0.952, respectively. The fitted lines did not pass through the origin, suggesting the presence of multiple adsorption mechanisms [28].

The adsorption of phosphate onto TCAH from solutions with initial concentrations ranging from 2 to 100 mg P/L at temperatures of 5 °C, 15 °C, and 25 °C is illustrated in Figure 4b. The adsorption capacity increased gradually with the increase in phosphate concentration and temperature. The maximum adsorption capacity of TCAH reached 199.80 mg P/g at 25 °C.

Langmuir, Freundlich, and Redlich–Peterson adsorption isotherm models were used to describe the adsorption at different equilibrium concentrations (see Text S2 in the Supplementary Material). As presented in Figure 4b and Table S3, the phosphate adsorption onto TCAH was more consistent with the Redlich–Peterson adsorption isotherm model. The Redlich–Peterson model was commonly used to assess the reliability of the Langmuir model. The g values of TCAH were 0.83, 0.85, and 0.84 at 5, 15, and 25 °C, respectively, all falling between 0 and 1. This indicated that Langmuir model partially influenced the adsorption process but cannot determine it [29].

The thermodynamic parameters were calculated to understand the nature of the phosphate adsorption onto TCAH (Text S3 in the Supplementary Material). The good linearity between lnKc and 1/T obtained from the thermodynamic calculations (R^2^ = 0.981, Figure S2) indicated an accurate estimation of the thermodynamic parameters. As listed in Table S4, the Gibbs free energy changes (∆G°) for phosphate adsorption onto TCAH at all three temperatures were negative, indicating that the reaction was spontaneous [30]. The positive values of both enthalpy change (∆H°) and entropy change (∆S°) suggested that the adsorption reaction was endothermic and that increasing temperature enhanced the disorder of the adsorbed species [31,32].

### 2.4. Influence of pH and Coexisting Ions

**Figure 5:**
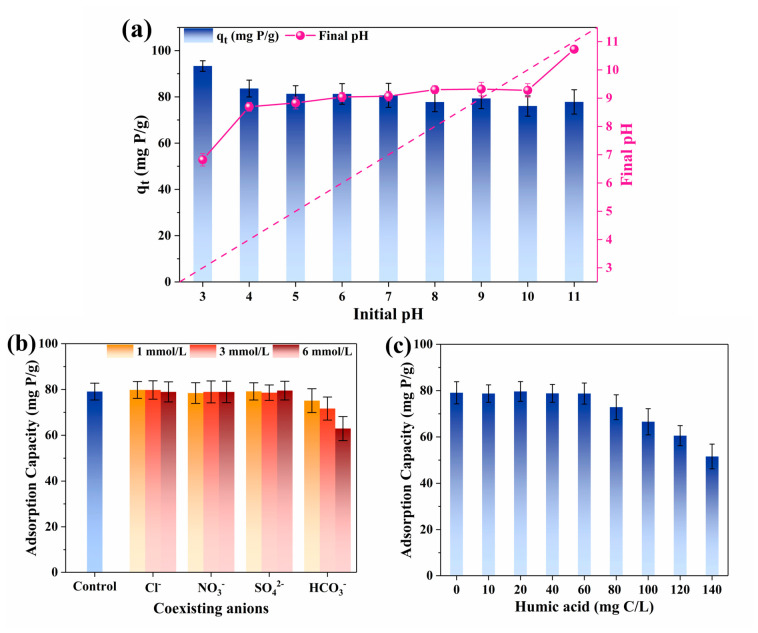
Effect of pH (**a**), coexisting anions (**b**), and humic acid (**c**) on phosphate adsorption by TCAH. (Adsorption dose = 0.1 g/L, C_0_ = 10 mg P/L, T = 25 °C, contact time = 24 h).

As shown in Figure 5a, the phosphate adsorption capacity of TCAH exhibited only a slight decrease with increasing initial pH. The adsorption capacity was relatively higher at an initial pH of 3.0 (93.32 mg P/g), while remaining around 80 mg P/g at other pH values. This result indicated that TCAH maintained high phosphate removal performance in solutions across a range of pH values, thus demonstrating considerable potential for practical applications. Compared with unmodified CAH_10_ [15], the influence of pH on the phosphate adsorption of TCAH was significantly reduced, which may be attributed to the different adsorption mechanisms between TCAH and CAH_10_ [33].

Figure 5b illustrates the effects of several anions on phosphate adsorption. The presence of Cl^−^, NO_3_^−^, and SO_4_^2−^, which are commonly found in wastewater treatment, generally did not affect the adsorption capacities. However, when the molar concentration of HCO3^−^ reached about 20 times that of phosphate (6 mmol/L), the adsorption capacity decreased to 62.93 mg P/g. The affinity of HCO_3_^−^ for binding with Ca and Al cations may be the primary reason for the negative effect on phosphate adsorption [34].

The influence of humic acid on phosphate adsorption is presented in Figure 5c. When the humic acid concentration increased to 60 mg C/L, no significant change in adsorption capacity was observed, indicating that low concentrations of humic acid had a negligible effect on the adsorption. As the concentration was further increased to 140 mg C/L, the phosphate adsorption capacity decreased by approximately 35%. The adsorption capacity remained at 51.56 mg P/g, although humic acid may compete with phosphate for adsorption sites [11]. TCAH exhibited relatively high selectivity toward phosphate, which is favorable for its practical application in real wastewater treatment.

### 2.5. Possible Phosphate Removal Mechanism

In this study, CAH_10_ was synthesized via the hydration of pure calcium aluminate, while TCAH was obtained by the thermal modification of CAH_10_ at 120 °C. The hydration process is crucial in the formation and structural transformation of calcium aluminate-based materials [35]. Meanwhile, the crystal structure and surface morphology of TCAH may also be influenced by rehydration during phosphate adsorption in aqueous solutions. Therefore, TCAH samples rehydrated in deionized water were tested for comparisons. To elucidate the phosphate removal mechanism, the pristine TCAH, TCAH before and after phosphate adsorption, and rehydrated TCAH were systematically characterized using SEM, XRD, FTIR, and XPS.

**Figure 6:**
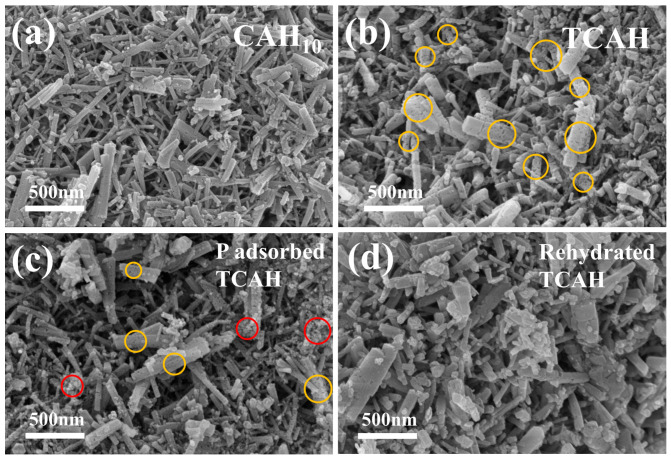
SEM images of pristine CAH_10_ (**a**), TCAH (**b**), phosphate-adsorbed TCAH (**c**), and rehydrated TCAH (**d**). Yellow circle: pores and cracks on the crystal surface; red circle: particles formed on the crystal surface.

Figure 6 presents the SEM images of pristine CAH_10_, TCAH, phosphate adsorbed TCAH, and rehydrated TCAH. As shown in Figure 6a, unmodified CAH_10_ exhibited intact prismatic crystals. In contrast, some pores were observed on the prismatic crystals of TCAH in Figure 6b (marked by yellow circles). After phosphate adsorption (Figure 6c), these pores remained visible, and the prismatic morphology did not revert to the more intact form of pristine CAH_10_. Furthermore, new particles were formed on the surface of the prisms in Figure 6c (marked by red circles, images at higher magnification are provided in Figure S3 in the Supplementary Material), which may correspond to surface precipitates formed during phosphate adsorption.

Figure 6d shows the SEM image of rehydrated TCAH. Compared with TCAH before and after phosphate adsorption, the pores on the crystal surface of rehydrated TCAH were significantly reduced. Rehydrated TCAH exhibited a more complete prismatic morphology, which suggested that TCAH underwent rehydration and recovered the prismatic crystal structure in phosphate-free deionized water. This phenomenon is similar to the “memory effect” exhibited by thermally modified layered double hydroxides (LDHs) [36,37]. In contrast to the rehydrated TCAH, the phosphate-adsorbed TCAH failed to recover the prismatic crystal morphology, as the presence of phosphate inhibited the rehydration process.

EDS analyses were performed on two distinct morphological features of TCAH after phosphate adsorption: the prismatic crystal regions (yellow circles in Figure 6c) and the precipitated particles (red circles in Figure 6c). The resulting EDS spectra and elemental distributions are shown in Figure S4 in the Supplementary Material. The adsorbed phosphorus was relatively uniformly distributed on the prismatic crystal surfaces, with an atomic percentage of 6.38% in the analyzed region. In contrast, the phosphorus content on the surface of the precipitated particles (Figure S4d–f) was relatively higher, with an atomic percentage of 9.91%. The EDS spectra confirmed the successful adsorption of phosphate in two different forms.

**Figure 7:**
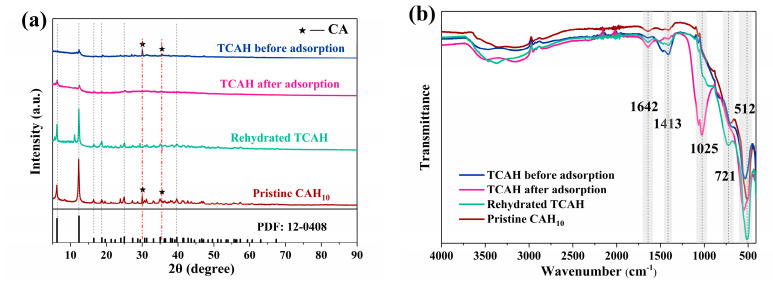
XRD patterns (**a**) and FTIR spectra (**b**) of pristine CAH_10_, TCAH before/after phosphate adsorption, and rehydrated TCAH.

As presented in Figure 7a, TCAH exhibited very low crystallinity compared with CAH_10_ before thermal modification (PDF# 12-0408). After phosphate adsorption, only weak characteristic peaks of CAH_10_ were observed, and the crystallinity remained extremely low. The phosphate-loaded TCAH failed to recover the pristine CAH_10_ crystal structure, which is consistent with the porous surface morphology observed in Figure 6c. In addition, no other phosphorus-containing crystal structures were detected after phosphate adsorption, which may be attributed to the low crystallinity of the newly formed surface precipitates (red circles in Figure 6c), rendering them undetectable by XRD [38]. However, when TCAH was rehydrated in deionized water, the XRD pattern confirmed the recovery of the CAH_10_ crystal structure with high diffraction peak intensities, which was consistent with the relatively intact prismatic morphology shown in Figure 6d. The formation of different crystal structures by TCAH in phosphate solution and deionized water confirmed that phosphate adsorption significantly influenced the rehydration process of TCAH.

Figure 7b shows the FTIR spectra of TCAH before and after phosphate adsorption, as well as after rehydration. The absorption band at approximately 1642 cm^−1^ was attributed to the stretching vibration of hydroxyl groups, which may be associated with adsorbed H_2_O, hydroxides, or hydrates [25,36]. The absorption peaks observed at around 1413 cm^−1^ and 512 cm^−1^ were ascribed to Al–H stretching vibrations [24] and (Ca, Al)–O lattice vibrations [13,39], respectively. These three absorption bands were present in all the tested samples, indicating that the (Ca, Al)-O structure was retained after thermal modification and phosphate adsorption. The absorption peak at 721 cm^−1^ could be assigned to the characteristic vibration of Al-OH bond [40,41]. A new absorption peak appeared at 1025 cm^−1^ for TCAH after phosphate adsorption, corresponding to ν3 band vibration of HPO_4_^2-^ or H_2_PO_4_^-^ [42], which confirmed the successful phosphate uptake.

**Figure 8:**
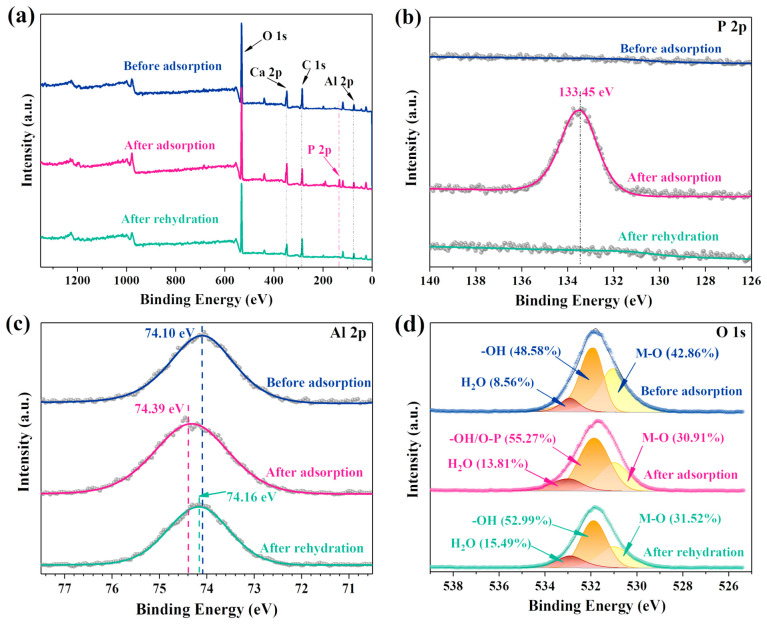
XPS spectra of TCAH before adsorption, after adsorption, and after rehydration treatment. (**a**) wide scan, (**b**) P 2p, (**c**) Al 2p, and (**d**) O 1s.

Figure 8 presents XPS spectra of TCAH before adsorption, after adsorption, and after rehydration treatment. In Figure 8a, a new P 2p peak appeared after adsorption, confirming the successful uptake of phosphate. The high-resolution P 2p spectrum in Figure 8b shows that the P 2p peak of TCAH after phosphate adsorption was located at a binding energy of 133.45 eV, which was lower than that of pure KH_2_PO_4_ (approximately 134.0 eV), indicating that the adsorbed phosphate formed new chemical bonds [22]. From the high-resolution Al 2p spectra in Figure 8c, the Al 2p binding energy peaks of TCAH before phosphate adsorption and after rehydration were located at 74.10 eV and 74.16 eV, respectively, which are relatively close to each other. In contrast, the Al 2p peak of TCAH after phosphate adsorption shifted to 74.39 eV, suggesting the possible formation of Al-O-P complexes during the phosphate adsorption process [19], whose binding energy differs substantially from that of the original Al-O structure.

The high-resolution O 1s spectrum (Figure 8d) was fitted into three peaks at 530.99, 531.88, and 532.92 eV, which were assigned to M-O (M = Al, Ca), -OH, and adsorbed H2O, respectively [43,44]. Comparing the O 1s spectra of TCAH before and after adsorption, phosphate adsorption reduced the proportion of M-O from 42.81% to 30.91%, while the proportions of -OH, O-P, and H_2_O increased substantially. The changes in the three peak area ratios may result from the combined effects of O-P bond formation during phosphate adsorption and the rehydration of TCAH [42]. After rehydration, the proportion of the H_2_O increased to 15.49%, indicating that the hydration process significantly enhanced the adsorbed H_2_O compared to TCAH before and after phosphate adsorption. The XPS results further demonstrated that the adsorption process of TCAH in phosphate solution involved not only the inner-sphere complexation but also the rehydration of TCAH. Meanwhile, the presence of phosphate in solution influenced the rehydration, which was consistent with the SEM and XRD analyses.

TCAH can be rehydrated in deionized water, restoring the CAH_10_ crystal structure disrupted during thermal modification. This phenomenon is similar to the “memory effect” observed in thermally modified Ca-Al layered double hydroxides (LDHs) during anion adsorption [37,45]. However, in phosphate solution, the adsorption of phosphate by TCAH restricted the alteration and recovery of its crystal structure, providing a useful basis for elucidating the phosphate adsorption mechanism.

In phosphate solution, some Ca and Al ions on the surface of TCAH were leached out (see Figure S5 in the Supplementary Material), followed by reprecipitation and recrystallization. The leached Ca^2+^ and Al^3+^ preferentially react with phosphate to form amorphous precipitates (Figure 7a and Figure S4). These precipitates consume the cations needed to rebuild the CAH_10_ lattice and create a surface barrier, thereby arresting recrystallization. Consequently, the amorphous precipitate phase becomes the dominant sink for phosphate removal, immobilizing phosphate in a stable non-crystalline form. Previous studies have shown that the hydration process of calcium aluminate can be influenced by the addition of phosphate [46,47], resulting in the formation of amorphous calcium aluminum phosphate hydrates [48]. Similarly, no crystalline P-containing precipitates were detected by XRD in this study, confirming that the phosphate adsorption mechanism involved the formation of amorphous P-containing precipitates. Additionally, the characterization results of surface functional groups and chemical bonds before and after phosphate adsorption also indicate the presence of inner-sphere complexation during phosphate uptake by TCAH [15].

Based on the above results, SEM-EDS (Figure 6c and Figure S4) showed that new particles on TCAH after adsorption contain higher P (9.91 at%) than the prismatic surfaces (6.38 at%), and rehydrated TCAH recovered its prismatic morphology only in phosphate-free water (Figure 6d), confirming that phosphate stabilized amorphous precipitates. XRD (Figure 7a) detected no crystalline P-containing phases, and the weak CAH_10_ peaks further supported the formation of amorphous precipitates. FTIR (Figure 7b) confirmed phosphate uptake, while the absence of crystalline phosphate peaks also indicated inner-sphere complexation. Finally, XPS (Figure 8c,d) showed an Al 2p shift from 74.10 to 74.39 eV, suggesting Al–O–P complexes, and O 1s exhibited decreased M–O with increased –OH/O–P, consistent with inner-sphere complexation mechanism.

**Figure 9:**
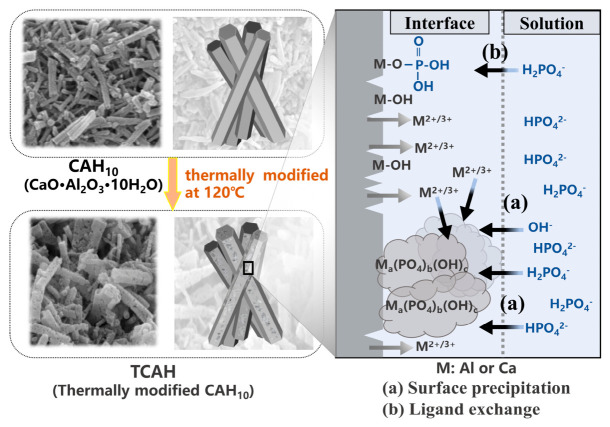
Schematic diagram for the mechanism of phosphate immobilization on TCAH.

In summary, the adsorption of phosphate by TCAH was governed by the combined effect of amorphous precipitation and inner-sphere complexation. Phosphate removal was primarily governed by a precipitation process, in which the released Ca and Al ions combined with phosphate to form amorphous precipitates. Meanwhile, ligand exchange between phosphate groups in solution and hydroxyl groups on the TCAH surface resulted in the formation of inner-sphere complexes. A schematic diagram of the proposed immobilization mechanism is shown in Figure 9.

### 2.6. Application Potential

Table 1 presents the phosphate adsorption performance of TCAH and recently reported metal (hydr)oxide-based adsorbents. Compared with the pristine CAH_10_, thermal modification significantly enhanced the phosphate adsorption performance of TCAH. Although the production of the calcium aluminate raw material required high temperatures (1000 °C and 1400 °C). Nevertheless, calcium aluminate is the primary component of aluminate cement and is already commercially available on a large scale. Moreover, the required thermal modification temperature for TCAH (120 °C) was much lower than that of other adsorbents reported previously, such as LDHs (300–500 °C) [37], attapulgite (700 °C) [49], waste biomass (500 °C) [50], red mud (600–1000 °C) [51], and construction waste (800 °C) [52]. The readily available raw material and low modification temperature endowed TCAH with obvious advantages in simplifying preparation procedures and reducing energy consumption, highlighting its potential for industrial-scale production and practical application.

As shown in Table 1, the maximum phosphorus adsorption capacity (*q_max_*) of TCAH reached 199.80 mg P/g, which was higher than that of most rare-earth metal composites listed in the table. Additionally, TCAH exhibited an excellent initial adsorption rate. The adsorption capacity of TCAH reached 54.53 mg P/g within the first hour of adsorption, which was comparable to most adsorbents included in the table.

The P-loaded TCAH was obtained with a maximum P content of 199.80 mg P/g, equivalent to 45.78% when expressed as P_2_O_5_. The P content on TCAH was higher than that of the commonly seen natural P ore (35% P_2_O_5_) [56], indicating the potential of TCAH to be utilized as a P-containing raw material. Benefiting from its high adsorption capacity and fast adsorption kinetics, TCAH showed promising prospects for P recovery from high-strength phosphate wastewater, such as sludge digestion liquor, aquaculture wastewater and industrial wastewater. Meanwhile, since calcium aluminate itself is the primary component of calcium aluminate cement, the exposed TCAH—if not used for phosphorus recovery—could be considered for reuse as a construction material [35,47].

The phosphate removal efficiency of TCAH for sludge P-releasing liquor is presented in Figure S6 in the Supplementary Material. After anaerobic digestion and acidification, the phosphate concentration of the obtained sludge P-releasing liquor reached about 134 mg P/L (detailed parameters are provided in Table S5). Given the ion release behavior, TCAH should be applied similarly to chemical agents used for phosphate removal, with the dosage controlled to avoid secondary pollution. When TCAH was applied at a dosage of 5 g/L, the phosphorus removal efficiency exceeded 80% within 0.5 h, and nearly reached 100% after 6 h, suggesting its high potential for practical applications.

## 3. Materials and Methods

### 3.1. Materials

All chemicals were of analytical grade and used as received without further purification in this study. CaCO_3_, Al_2_O_3_, KH_2_PO_4_, NaOH, NaCl, NaNO_3_, Na_2_SO_4_, and NaHCO_3_ were obtained from Kermel Chemical Regent Co., Ltd., Tianjin, China.

Calcium aluminate (CA) was first prepared via a solid-state reaction following the method described by Klaus et al. [57]. CaCO_3_ and Al_2_O_3_ were mixed at a molar ratio of 1:1, then heated in a quartz crucible at a rate of 10 °C/min to 1000 °C and calcined for 4 h to remove CO_2_. Subsequently, the temperature was raised to 1400 °C and maintained for 6 h. After cooling, the product was ground and homogenized using a vibratory mill. The calcination and grinding procedures were repeated once as described. The crystal structure of the final product was confirmed by X-ray diffraction analysis.

Calcium aluminate decahydrate (CAH_10_) was synthesized by hydration of CA [15]. In brief, CA was mixed with degassed deionized water at a water-to-solid mass ratio of 10:1 in polypropylene centrifuge vials. The vials were cured at 5 ± 1 °C for 7 days with periodic shaking at approximately 200 r/min to prevent agglomeration. The solid sample was then separated by centrifugation, washed several times with deionized water, and freeze-dried for 24 h. The resulting product was ground, sieved through 160 mesh, and stored in airtight bags at 5 ± 1 °C.

For thermal modification, the as-prepared CAH_10_ was heated from room temperature to the target temperature at a rate of 10 °C/min, followed by thermal treatment for 1 h. The thermal modification temperatures were set at 120 °C, 220 °C, 300 °C, 400 °C, and 600 °C, respectively. After natural cooling, the samples were stored in airtight containers for further experiments.

### 3.2. Adsorption Experiments

#### 3.2.1. Determination of Adsorption Capacity

The phosphate adsorption capacity was determined through batch adsorption experiments. Phosphate solutions were prepared by dissolving KH_2_PO_4_ with an initial concentration at 10 mg P/L. The ionic strength of the phosphate solution was adjusted to 0.01 mol/L using NaCl, and the initial pH was adjusted to 7.0 ± 0.1 using 0.1 mol/L NaOH or HCl solutions. All regents were of analytical grade. All adsorption tests were conducted in triplicate to obtain mean values and standard deviations.

Adsorbents were mixed with the phosphate solution at a dosage of 0.1 g/L in conical flasks. The flasks were placed in a constant-temperature shaker and agitated at 25 °C and 180 r/min. After adsorption, samples were collected using a syringe, filtered through a 0.45 μm membrane filter, and subsequently analyzed for phosphate concentration.

The adsorption capacity at time *t* (h), denoted as *q_t_* (mg P/g), was calculated using Equation (1):

(1) $$q_{t}=\frac{C_{0}-C_{t}}{m}\times V$$

where *m* (g) is the mass of the adsorbent, *C*_0_ (mg P/L) is the initial phosphate concentration, *C_t_* (mg P/L) is the phosphate concentration at adsorption time *t* (h), and *V* (L) is the solution volume. When adsorption reached equilibrium, the equilibrium adsorption capacity *q_e_* (mg P/g), was calculated using Equation (2):

(2) $$q_{e}=\frac{C_{0}-C_{e}}{m}\times V$$

where *C_e_* (mg P/L) is the phosphate concentration at equilibrium.

#### 3.2.2. Adsorption Kinetics, Isotherms, and Thermodynamics Studies

For the adsorption kinetics experiments, the adsorbent was added to a phosphate solution with a concentration of 10 mg P/L at a dosage of 0.1 g/L. Then, the mixtures were agitated for 7 days at 25 °C and 180 r/min. Samples were collected at predetermined intervals during this period to determine the phosphorus concentration.

For the adsorption isotherm and thermodynamic studies, experiments were conducted at 5 °C, 15 °C, and 25 °C, respectively. A constant adsorbent dosage of 0.1 g/L and initial phosphate concentrations ranging from 2 to 100 mg P/L were used. The mixtures were agitated for 60 h at 180 r/min. The phosphate concentrations were measured to calculate the equilibrium adsorption capacities, from which the adsorption isotherms were derived.

#### 3.2.3. Determination of Adsorption Selectivity

To investigate the effect of pH on adsorption, the initial pH of the phosphate solution was adjusted to values ranging from 3.0 to 11.0. Then, the adsorption capacity and the final pH of the solution were measured after equilibrium was reached.

To examine the influence of coexisting anions, phosphate solutions containing Cl^-^, SO_4_^2-^, NO_3_^-^, or HCO_3_^-^ anions were prepared using NaCl, NaNO_3_, Na_2_SO_4_, and NaHCO_3_, respectively. The initial phosphate concentration was maintained at 10 mg P/L (approximately 0.3 mmol/L). The molar concentrations of each coexisting anion were set at 1, 3, and 6 mmol/L, corresponding to approximately 3, 10, and 20 times the molar concentration of phosphate, respectively. To assess the impact of dissolved organic matter (DOM), humic acid (5–100 mg C/L, in dissolved organic carbon) was used as a representative of DOM.

#### 3.2.4. Rehydration of TCAH

To explore the adsorption mechanism, TCAH was rehydrated for comparison with the phosphate adsorption process. TCAH was added at a dosage of 0.1 g/L to a conical flask containing CO_2_-free deionized water. The mixture was sealed and placed in a thermostatic shaker at 25 °C and 180 r/min for 24 h. Subsequently, the solid sample was separated by centrifugation and freeze-dried for 24 h to obtain the rehydrated TCAH. Meanwhile, samples were taken at predetermined time intervals, and the concentrations of calcium and aluminum ions in the solution were measured.

#### 3.2.5. Practical Application

TCAH was used to treat the phosphorus-releasing liquor derived from excess sludge of a wastewater treatment plant to evaluate its practical application performance. The sludge samples were collected from the secondary sedimentation tank of a wastewater treatment plant (A^2^/O process) in Nanyang City, Henan Province, China. The sludge was settled at 4 °C for 2 h, after which the supernatant was discarded. The residual sludge was then subjected to anaerobic treatment at 25 °C for 72 h. Finally, the pH was adjusted to 2.0 using 2 mol/L HCl and maintained for 4 h. After centrifugation, the phosphorus-releasing liquor was obtained. TCAH was added to the phosphorus-releasing liquor at dosages of 2 and 5 g/L, respectively. Samples were taken at 1, 2, 4, 6, and 12 h to determine the phosphate concentration, thereby assessing the actual phosphate removal efficiencies.

### 3.3. Characterizations and Analysis

The phosphate concentration was determined using the molybdenum-blue method. The surface micromorphology of the materials was observed using a scanning electron microscope (SEM, JSM-6510LV, JEOL, Tokyo, Japan) equipped with an energy dispersive spectroscopy (EDS) detector. Thermal gravimetric analyses were conducted by a thermal gravimetric analyzer (STA 2500, Netzsch, Bavaria, Germany) with a temperature range of 40–900 °C and a heating rate of 5 °C/min. The specific surface area and pore size distribution were determined based on the nitrogen adsorption/desorption data using a surface area analyzer (ASAP 2460, Micromeritics, Norcross, GA, USA). The crystalline structure was determined using an X-ray diffractometer (XRD, Ultima IV, Rigaku, Tokyo, Japan) with a Cu Kα radiation and a scanning range (2θ) of 5–90°. The Fourier transform infrared spectra (FTIR) were collected on a Nicolet IS50 FTIR spectrometer (Thermo Fisher Scientific, Waltham, MA, USA) with a scanning range of 400–4000 cm^−1^. X-ray photoelectron spectroscopy (XPS) spectra were obtained by a XPS spectrometer system (ESCALAB Xi+, Thermo Fisher Scientific, Waltham, MA, USA), with C 1s (284.8 eV) as the reference.

## 4. Conclusions

TCAH was prepared by thermal modification of CAH_10_ at a relatively low temperature of 120 °C as a novel phosphate adsorbent. The thermal treatment reduced the crystallinity of CAH_10_, enhanced its porosity, and induced the formation of amorphous calcium aluminate phases. Hence, the phosphate adsorption performance was significantly improved with a maximum adsorption capacity of 199.80 mg P/g. The phosphate adsorption on TCAH followed pseudo-second-order kinetic and Redlich–Peterson isotherm models well, with stable efficiency over a wide pH range (3.0–11.0) and high phosphate selectivity. Phosphate uptake was governed by a dual mechanism involving amorphous precipitation and inner-sphere complexation. TCAH achieved near-complete phosphate removal from sludge P-releasing liquor at a dosage of 5 g/L, demonstrating that TCAH is a promising candidate for efficient phosphate removal and recovery from wastewater.

## Figures and Tables

**Table 1:** Comparison of the phosphate adsorption performance with various metal (hydr)oxide related adsorbents.

Adsorbents	*q_max_* (mg P/g)	*C*_0_ (mg P/L)	Contact Time (h)	Ref.
TCAH	199.80	2–100	24	this study
Pristine CAH_10_	89.58	2–100	60	[15]
Modified La-MOF	131.52	5–200	12	[5]
Zr-MOFs(MOF-808)	65.45	5–100	24	[6]
ZIF-8@aminated PAN-PVP	79.30	1–200	1	[7]
Lanthanum-functionalised forestry waste biochar	112	1–500	24	[8]
La-Zr co-modified hydrogel beads	88.97	0–100	20	[9]
La/Al engineered bentonite composite	83.32	50–200	24	[19]
Goethite dispersed corn straw-derived biochar	57.39	17.5–1400	48	[53]
Zirconium-loaded Ca-montmorillonite	22.37	1–50	24	[54]
Zirconium oxide-intercalated sodium montmorillonite scaffold	65.35	50–100	10	[55]

## Data Availability

The original contributions presented in this study are included in the article. Further inquiries can be directed to the corresponding author.

## Supplementary Materials

The following supporting information can be downloaded at: https://www.mdpi.com/article/10.3390/molecules31122174/s1. Figure S1: FTIR spectra of pristine CAH_10_ and thermally modified CAH_10_; Figure S2: The plot of lnKC versus 1/T; Figure S3: SEM image of TCAH after phosphate adsorption; Figure S4: SEM images (a,d), EDS spectra (b,e), and EDS mapping (c,f) of TCAH after phosphate adsorption; Figure S5: Concentrations of calcium and aluminum ions released from TCAH in deionized water; Figure S6: Phosphate removal efficiency of TCAH for sludge P-releasing liquor; Table S1: The specific surface area and pore characteristics of CAH_10_ at different thermally treated temperatures; Table S2: Kinetic parameters for phosphate adsorption on TCAH; Table S3: Adsorption isotherm parameters for phosphate adsorption on TCAH; Table S4: The thermodynamic parameters for the adsorption of phosphate on TCAH; Table S5: Physicochemical properties of the liquid phase of the sludge after P release treatment; Text S1: Kinetic models; Text S2: Isotherm models; Text S3: Adsorption thermodynamics.

## Abbreviations

The following abbreviations are used in this manuscript:

TCAH	Thermally modified calcium aluminate decahydrate
CAH_10_	Calcium aluminate decahydrate, CaO·Al_2_O_3_·10H_2_O
C_3_AH_6_	3CaO·Al_2_O_3_·6H_2_O
