# Supplementary material for: Enhanced Phosphate Capture by Thermally Modified Calcium Aluminate Decahydrate: Optimization, Performance and Mechanism
Source: Molecules. 2026 Jun 21;31(12):2174. doi: 10.3390/molecules31122174 (PMC13304520; doi:10.3390/molecules31122174)
Supplement: Supplementary file 1 [file molecules-31-02174-s001.zip › molecules-4353083-supplementary.pdf]

Supporting Information for

Enhanced Phosphate Capture by Thermally Modified Calcium Aluminate Decahydrate: Optimization, Performance and Mechanism

Peng Cheng [1,2,*], Ruixiang Wang [1,2], Yu Liu [1,2], Yu Shang [1,2], Lei Yang [3] and Yong-Xiang Ren [3]

[1] Nanyang Key Laboratory of Water Pollution Control and Solid Waste Resource Recovery, Nanyang Institute of Technology, Nanyang 473004, China; wangruixiang35@163.com (R.W.); gps_liuyu@126.com (Y.L.); yshangnyist@163.com (Y.S.)
[2] School of Civil Engineering, Nanyang Institute of Technology, Nanyang 473004, China
[3] Shaanxi Key Laboratory of Environmental Engineering, Xi'an University of Architecture and Technology, Xi'an 710055, China; yangleigps@xauat.edu.cn (L.Y.); ryx@xauat.edu.cn (Y.-X.R.)
* Correspondence: gps_pengcheng@126.com

This file includes:

Text S1 to S3

Figure S1 to S6

Table S1 to S5

Total pages: 8

Text S1. Kinetic models

Nonlinear forms of several adsorption kinetics and isotherm models were employed to describe adsorption data and evaluate the adsorption performance in this study.

The pseudo-first-order, pseudo-second-order, Elovich, and intra-particle diffusion kinetics models can be expressed in Equation (S1), Equation (S2) Equation (S3), and Equation (S4), respectively.

$$q_t = q_e(1 - e^{-k_1 t}) \tag{S1}$$

$$q_t = \frac{q_e^2 k_2 t}{1 + k_2 q_e t} \tag{S2}$$

$$q_t = \frac{1}{\beta_E} \ln(1 + \alpha_E \beta_E t) \tag{S3}$$

$$q_t = k_3 t^{\frac{1}{2}} + \mathrm{C} \tag{S4}$$

Where q_t (mg/g) denotes the adsorption capacity at any given time t (h), q_e (mg/g) represents the adsorption capacity at equilibrium, k_1 (1/h), k_2 (g/(mg·h)), and k_3 (mg/(g·h$^{1/2}$)) are the corresponding rate constants of the pseudo-first-order, pseudo-second-order and intra-particle diffusion models, α_E (mg/(g·h)) is the initial rate constant and β_E (g/mg) is the desorption constant in Elovich model, C (mg/g) is a constant associated with the thickness of the boundary layer in the intra-particle diffusion model.

Text S2. Isotherm models

Langmuir, Freundlich and Redlich-Peterson adsorption isotherm models are expressed in Equation (S5), Equation (S6) and Equation (S7), respectively:

$$q_e = \frac{Q_m k_L C_e}{1 + k_L C_e} \tag{S5}$$

$$q_e = k_F C_e^{\frac{1}{n}} \tag{S6}$$

$$q_e = \frac{k_{RP} C_e}{1 + \alpha_{RP} C_e^g} \tag{S7}$$

q_e (mg/g) is the adsorption capacity at equilibrium, Q_m (mg/g) is the maximum adsorption capacity, k_L (L/mg) is the Langmuir adsorption constant, k_F

((mg/g)(L/mg)$^{1/n}$) and $1/n$ (dimensionless) are the Freundlich adsorption constants, k_{RP} (L/g) and α_{RP} (mg/L)$^{-g}$ are corresponding constants of Redlich-Peterson isotherm model, and g (dimensionless) is an exponent factor related to the adsorption intensity.

Text S3. Adsorption thermodynamics

The phosphate adsorption thermodynamics parameters were obtained through the following equations:

$$\Delta G^{o} = -RT \ln K_C \tag{S8}$$

$$\ln K_C = \frac{-\Delta H^{o}}{R} \times \frac{1}{T} + \frac{\Delta S^{o}}{R} \tag{S9}$$

Where ΔG^{o} (J/mol) is the Gibbs free energy, which is directly calculated from Equation (S8). ΔH^{o} (J/mol) and ΔS^{o} (J/(mol·K)) are the enthalpy change and entropy change, which are obtained from the slope and intercept of a plot of $\ln K_C$ versus 1/T (Equation (S9)), respectively. R is the universal gas constant (8.314 J/(mol·K)). The equilibrium constant K_C (dimensionless) was computed by using Equation (S10).

$$K_C = \frac{k_F \rho}{1000} \left(\frac{10^6}{\rho}\right)^{\left(1-\frac{1}{n}\right)} \tag{S10}$$

in which, ρ is the density of pure water (assumed as ~1.0 g/mL), k_F ((mg/g)(L/mg)$^{1/n}$) and $1/n$ (dimensionless) are the Freundlich adsorption constants.

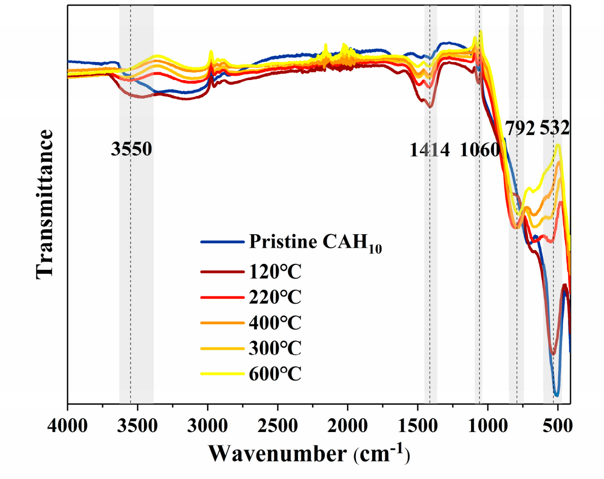

Figure S1. FTIR spectra of pristine CAH10 and thermally modified CAH10. (Modified temperature=120°C, 220°C, 300°C, 400°C and 600°C).

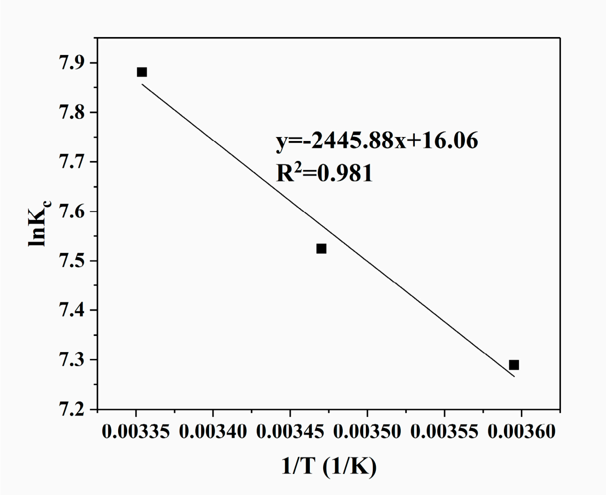

Figure S2. The plot of ln$\boldsymbol{K_C}$ versus 1/T.

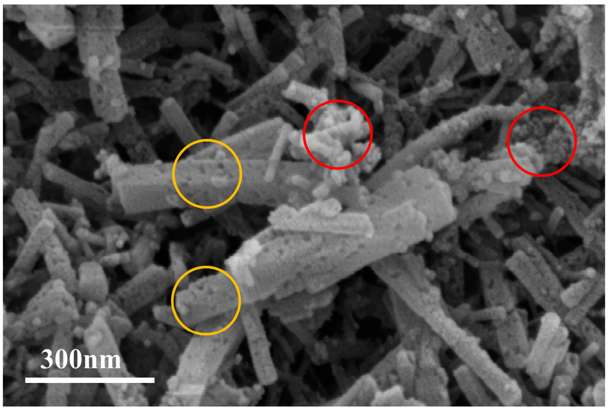

Figure S3. SEM image of TCAH after phosphate adsorption.

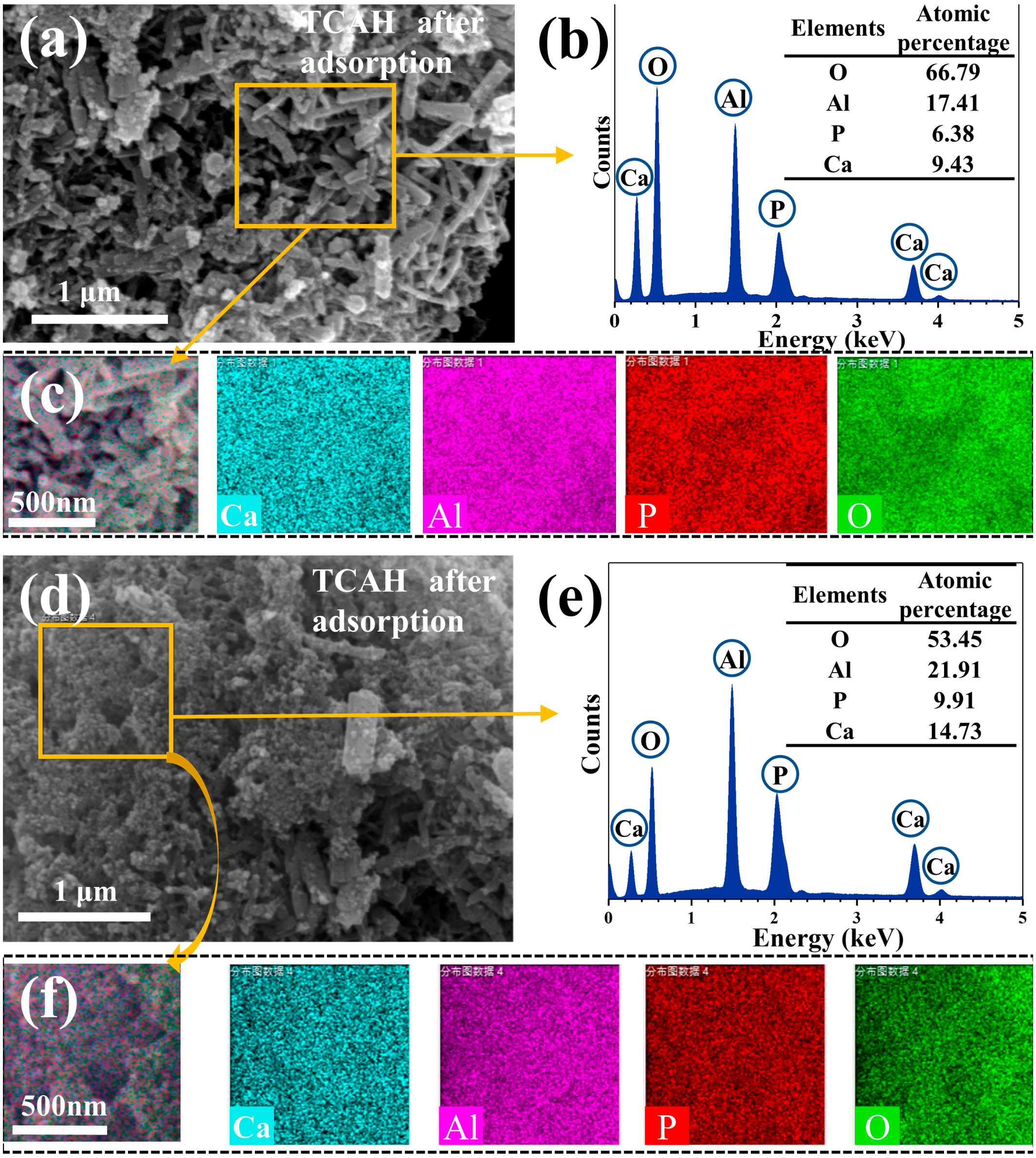

Figure S4. SEM images (a, d), EDS spectra (b, e), and EDS mapping (c, f) of TCAH after phosphate adsorption. (a, b, c): prismatic crystals, (d, e, f): aggregated particles.

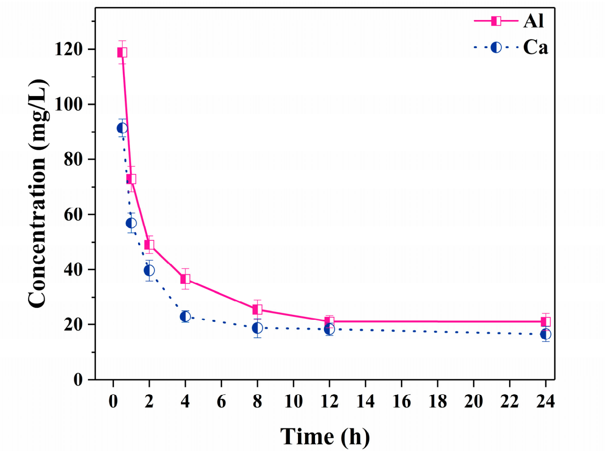

Figure S5. Concentrations of calcium and aluminum ions released from TCAH in deionized water (dosage: 0.1 g/L, temperature: 25°C).

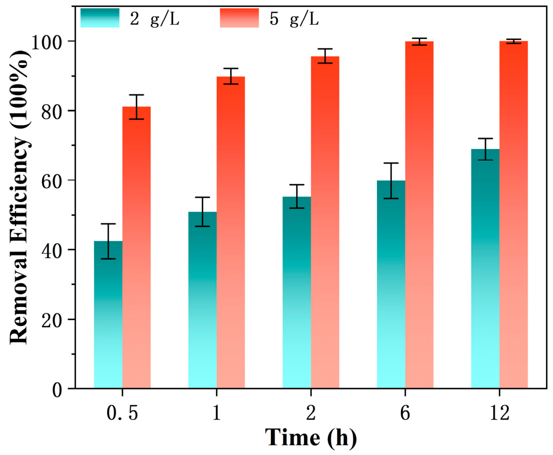

Figure. S6. Phosphate removal efficiency of TCAH for sludge P-releasing liquor.

Table S1

The specific surface area and pore characteristics of CAH10 at different thermally treated temperatures

Modified temperature	Specific pore volumes （cm^3/g）	BET surface area （m^2/g）	Average pore diameter （nm）
Pristine CAH10	0.196	37.34	20.34
120℃	0.198	31.44	25.17
220℃	0.202	27.35	29.50
300℃	0.204	23.96	34.03
400℃	0.206	24.94	36.21
600℃	0.205	21.88	37.56

Table S2

Kinetic parameters for phosphate adsorption on TCAH

Pseudo-first-order model		Pseudo-second-order model		Elovich model	
q_e (mg/g)	68.55	q_e (mg/g)	74.46	α(g/mg·h)	1017.18
k_1(1/h)	2.49	k_2 (g/mg·h)	0.042	β(g/mg)	0.10
R^2	0.907	R^2	0.965	R^2	0.939
χ^2	7.37	χ^2	2.78	χ^2	4.82

Table S3

Adsorption isotherm parameters for phosphate adsorption on TCAH.

Adsorption isotherm	Temperature (°C)		
parameters	25	15	5
Langmuir			
Q_m (mg/g)	182.32	161.26	125.07
K_L(L/mg)	0.62	0.43	0.47
R^2	0.950	0.964	0.927
χ^2	237.71	123.61	140.81
Freundlich			
K_F (mg/g)(L/mg)$^{1/n}$	72.84	58.58	46.29
1/n	0.24	0.25	0.25
R^2	0.967	0.962	0.958
χ^2	156.35	132.03	81.33
Redlich-Peterson			
K_{RP} （L/g）	382.90	162.80	159.46
α_{RP}（g/mg·h）	3.83	1.82	2.47
g	0.84	0.85	0.83
R^2	0.994	0.992	0.980
χ^2	34.40	31.00	45.92

Table S4

The thermodynamic parameters for the adsorption of phosphate on TCAH.

T (K)	ΔG^o (J/mol)	ΔH^o (J/mol)	ΔS^o (J/(mol×K))
278.15	-16855.64		
288.15	-18025.74	20335.05	133.52
298.15	-19533.83		

Table S5

Physicochemical properties of the liquid phase of the sludge after P release treatment

Parameters	Unit	Value
TP	mg/L	135
PO4-P	mg/L	134
COD	mg/L	258.75
TN	mg/L	36.75
NH4-N	mg/L	28.84
Fe	mg/L	11.32
Ca	mg/L	96.15
Mg	mg/L	17.55
